# Implications of Isoprostanes and Matrix Metalloproteinase-7 Having Potential Role in the Development of Colorectal Cancer in Males

**DOI:** 10.3389/fonc.2018.00205

**Published:** 2018-06-07

**Authors:** Mahmood Rasool, Arif Malik, Ahmad Ashar Ghuman, Muhammad Abdul Basit Ashraf, Mahwish Arooj, Sulayman Waquar, Sara Zahid, Sumera Shaheen, Aamer Qazi, Muhammad Imran Naseer, Mazin A. Zamzami, Ayat Al-Ghafari, Othman A. Baothman, Mustafa Zeyadi, Nawal Helmi, Hani Choudhry, Mohammad Sarwar Jamal, Mohammed Hussein Al-Qahtani

**Affiliations:** ^1^Center of Excellence in Genomic Medicine Research, King Abdulaziz University, Jeddah, Saudi Arabia; ^2^Institute of Molecular Biology and Biotechnology, The University of Lahore, Lahore, Pakistan; ^3^Department of Biochemistry, Islam Medical College, Sialkot, Pakistan; ^4^University College of Medicine and Dentistry, The University of Lahore, Lahore, Pakistan; ^5^Centre for Research in Molecular Medicine, The University of Lahore, Lahore, Pakistan; ^6^Department of Biochemistry, Cancer Metabolism and Epigenetic Unit, Faculty of Science, King Abdulaziz University, Jeddah, Saudi Arabia; ^7^Center of Innovation in Personalized Medicine, King Abdulaziz University, Jeddah, Saudi Arabia; ^8^Cancer and Mutagenesis Unit, King Fahd Center for Medical Research, King Abdulaziz University, Jeddah, Saudi Arabia; ^9^King Fahd Medical Research Center, King Abdulaziz University, Jeddah, Saudi Arabia

**Keywords:** colorectal cancer, liver metastasis, matrix metalloproteinase-7, isoprostanes, lipid peroxidation

## Abstract

**Background:**

Colorectal cancer (CRC) is the third most common type of cancer and leading cause of death worldwide. Major risk factors involved in the development of CRC are increased dietary sources, genetics, and increasing age. Purpose of the study was to find the role of different variables in the progression of CRC.

**Methodology:**

50 blood samples from CRC patients and 20 samples from control were collected. Serum was separated from the blood by centrifugation. This serum was assessed for several antioxidants like superoxide dismutase (SOD), glutathione, glutathione peroxidase, glutathione reductase, catalase, vitamin A, C, and E, and pro-oxidants such as malondialdehyde, advanced oxidation protein products (AOPPs), and AGEs according to their respective protocols. Matrix metalloproteinase-7 (MMP-7) and isoprostanes were assessed by ELISA kits.

**Results:**

Lower levels of GSH (4.86 ± 0.78 vs 9.65 ± 1.13 μg/dl), SOD (0.08 ± 0.012 vs 0.46 ± 0.017 μg/dl), CAT (2.45 ± 0.03 vs 4.22 ± 0.19 μmol/mol of protein), and GRx (5.16 ± 0.06 vs 7.23 ± 0.36 μmol/ml) in the diseased group were recorded as compared with control. Higher levels of GPx (6.64 ± 0.19 mmol/dl) were observed in the subjects in comparison with control group (1.58 ± 0.30 mmol/dl). Highly significant decreased levels of vitamin A (0.81 ± 0.07 vs 2.37 ± 0.15 mg/ml), vitamin E (15.42 ± 1.26 vs 25.96 ± 2.19 mg/ml), and vitamin C (47.67 ± 7.69 vs 80.37 ± 10.21 mg/ml) were observed in the patients in contrast to control group. The reversal of antioxidants in later stages of CRC may be due to compensatory mechanisms in cancerous cells. The levels of MDA (nmol/ml) were also assessed, which shows significantly increased level in CRC patients as compared with control groups (3.67 ± 0.19 vs 1.31 ± 0.27). The levels of protein oxidation products [AGEs (2.74 ± 0.16 vs 0.84 ± 0.05 IU) and AOPPs (1.32 ± 0.02 vs 0.82 ± 0.07 ng/ml)] were significantly increased in subjects as compared with control. The levels of MMP-7 (64.75 ± 3.03 vs 50.61 ± 4.09 ng/ml) and isoprostanes (0.71 ± 0.03 vs 0.16 ± 0.02 ng/ml) were also analyzed. This shows that the levels of isoprostanes increased due to high lipid peroxidation mediate higher levels of MMP-7, which promotes development of CRC.

**Conclusion:**

Following study suggested that elevated oxidative and inflammatory status along with lipid peroxidation and matrix metalloproteinases are the chief contributors in the progression of CRC.

## Introduction

Cancer shows uncontrollable abnormal growth in which cancerous cells do not retaliate to normal growth controlling mechanisms and are less specialized than normal cells. Cancerous cells produce numbers of proteins that induce angiogenesis mediated by hypoxia-inducible factors thus, promote tumor proliferation (1). Worldwide, colorectal cancer (CRC) is the third most leading cause of death among all cancers which originates from the rapid division of epithelial cells that line the bowl. CRC has estimated prevalence of 1.3 million and about 0.7 million deaths annually, which accounts 9% deaths of all the cancers (2, 3). The oxidative insult occurs due to imbalance between syntheses and processing of reactive oxygen species (ROS) which react with cellular molecules such as lipids, proteins and DNA thus, modify gene expression resulting in cancer initiation and progression (4).

It has been revealed that Wnt signaling pathway, intracellular linkage, cytoskeleton stabilization, cell cycle regulation, and apoptosis are regulated by a tumor suppressor antigen-presenting cells (APC) gene. Mutation/inactivation of APC gene permits dysregulated transcription of oncogenes, as a result promote tumorigenesis (5). The APC gene mutations lead to the nuclear accumulation of beta catenin/TCF complex that acts as a transcriptional factor which in turn upregulates the matrix metalloproteinase-7 (MMP-7) expression (6). The matrix metalloproteinases (MMPs), zinc containing endopeptidases are responsible for metastasis of CRC, as they are important in the degradation of extracellular matrix containing elastin, gelatin, collagen, matrix glycoprotein, and proteoglycan. They are controlled through hormones, cytokines, and growth factors, and excreted *via* a number of connective tissues and pro-inflammatory cells, such as osteoblasts, macrophages, fibroblasts, endothelial cells, lymphocytes, and neutrophils (7). In CRC, the most common location for blood born metastasis is the liver, and MMP-7 plays a key role in it, as it is activated by MMP-3 and further activates MMPs 1, 2, and 9 (8). Overexpression of MMP-7 represents the early carcinogenesis of CRC and formation of adenoma from normal colorectal mucosa thus, it can be considered as a prognostic factor in the diagnosis of CRC (9).

The ROS-mediated oxidation of amino acids results in the production of advanced oxidation protein products (AOPPs), and in CRC, their levels are significantly increased (10). Isoprostanes are prostaglandins like compounds which are formed *in vivo* through the action of free radicals on arachidonic acid in the presence of an enzyme cyclooxygenase. It has been revealed that higher levels of isoprostanes may play a significant role in the pathogenesis of different cancers (11). The degree of lipid peroxidation is measured by the estimation of levels of malondialdehyde, and their raised levels suggest late stages of CRC (12). The objectives of this study were to assess the role of isoprostanes and MMP-7 in the development of CRC.

## Materials and Methods

This study (cross-sectional) was designed to evaluate the principle processes, which were involved in the association of isoprostanes levels with MMP-7 expression and evaluation of antioxidant activity in the male patients of CRC from the Jinnah Hospital, Lahore. Fifty male patients (50–70 years) of age group were included for the said project conducted during June 2015–August 2016. Informed consent was taken from all the subjects before being included in the study. All the experimental work was done at the Institute of Molecular Biology and Biotechnology (IMBB) and Center for research in Molecular Medicine (CRiMM), the University of Lahore. Twenty clinically apparent healthy individuals were included as controls. Protocols performed were according to the Research Ethical Committee of the IMBB and Center for research in Molecular Medicine (CRiMM), The University of Lahore. Five milliliters of venous blood samples were taken from the antecubital vein of each individual. Samples were centrifuged within 1 h of collection, serum were separated and stored at −70°C until assayed.

### Demographic Data

Patients with CRC were categorized in appropriate niches. Common niche for the disease remained *in situ* (cancer that yet not begun to invade the wall of colon or rectum), local (cancer that have grown into the wall of colon but not invaded the nearby tissues), regional (those cancer which spread through the wall of colon and invade the nearby tissues), and distant (cancer that shows malignancies such as it spreads to other parts of body such as liver or lung).

### Inclusion Criteria

Patients with confirmed diagnosis of CRC were included in the study.

### Exclusion Criteria

Subjects with prolonged history of taking drugs (including alcohol and cigarette) and pre-diagnosis medications (e.g., antiparkinsonian/antipsychotic) were excluded from this study. None of the controls were on any medication, history of chronic infections, malnutrition syndrome, depression, psychosis, or metabolic dysfunction (such as diabetes mellitus, liver diseases, and cancer) that could interfere with their oxidative status.

### Biochemical Analysis

Blood glutathione (GSH) was evaluated by the method of Moron et al. (13). GSH was estimated accordingly (the sum of reduced and oxidized glutathione of the sample) then unknown sample concentration was calculated by the help of linear equation generated with the help of several standards of glutathione. Catalase (CAT) was estimated by the method of Aebi (14). It was estimated by the help of taking absorbance at 230 nm by spectrophotometer. Then absorbance values of standards with known values were obtained to generate a standard curve. Lipid peroxidation in blood samples was estimated calorimetrically by measuring thiobarbituric acid reactive substances (TBARS) by the method of Ohkawa et al. (15). The levels of lipid peroxides were expressed as nanomoles per millimoles of TBARS. Superoxide dismutase (SOD) activity was determined by the method of Kakkar et al. (16). Glutathione reductase was evaluated by using method of David and Richard (17). Glutathione reductase (GRx) catalyzes the conversion of oxidized glutathione to reduced glutathione employing NADPH as a substrate. The amount of NADPH employed is a direct measure of enzyme activity. AOPPs were determined by spectrophotometer on a microplate reader (model MR 5000, Dynatech, Paris, France) and were calibrated with chloramine-T (Sigma, St. Louis, MO, USA) solutions that in the presence of potassium iodide absorb at 340 nm. The AGE-HSA used *in vitro* was prepared by incubating HSA (type V; Sigma; 50 mg/ml) with 500 mM glucose in PBS for 65 days at 37°C under sterile conditions. The AGE-pentosidine, as a marker of non-enzymatic glycation of proteins, was measured using a modification of the method described by Goldin et al. (18). Vitamin E was evaluated in samples by the Emmerie–Engel reaction as reported by Rosenberg (19). Tocopherols and carotenes are first extracted with xylene and read at 460 nm to measure carotenes. Vitamin A and vitamin C were determined by the methods of Bayfield and Cole (20) and Chinoy et al. (21). The levels of MMP-7 were detected by Human MMP-7 ELISA Kit. The levels of isoprostanes were detected by Human 8-epi-prostaglandin F2 alpha (8-iso-PGF2α) ELISA Kit.

### Statistical Analysis

The study design was prospective case–control, and the data were analyzed statistically by SPSS (V-16) and expressed as mean ± SD. ANOVA test was applied to analyze the results. Pearson’s correlation coefficients were used to correlate different variables. *p* < 0.05 is considered to be statistically significant.

## Results

**Figure 1:**
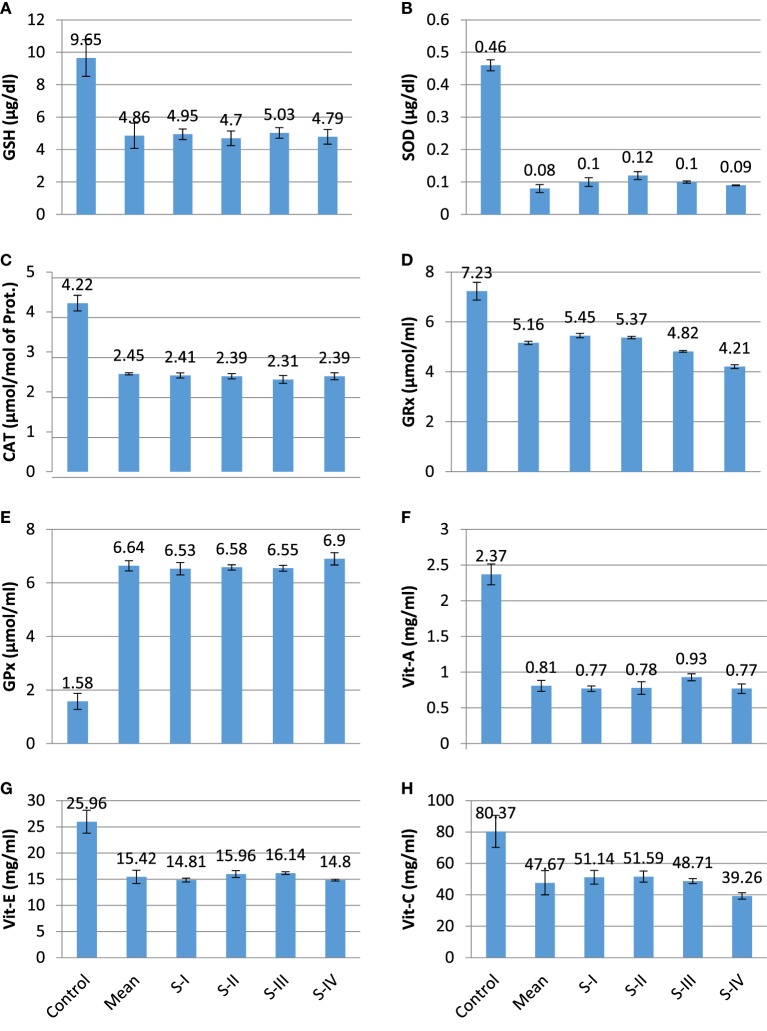
Profile of different prognostic variables in CA colorectal vs controls. **(A)** GSH (μg/dl); **(B)** superoxide dismutase (SOD) (μg/dl); **(C)** catalase (CAT) (μmol/mol of protein); **(D)** GRx (μmol/ml); **(E)** GPx (mmol/dl); **(F)** Vit A (mg/ml); **(G)** Vit E (mg/ml); and **(H)** Vit C (mg/ml).

**Figure 2:**
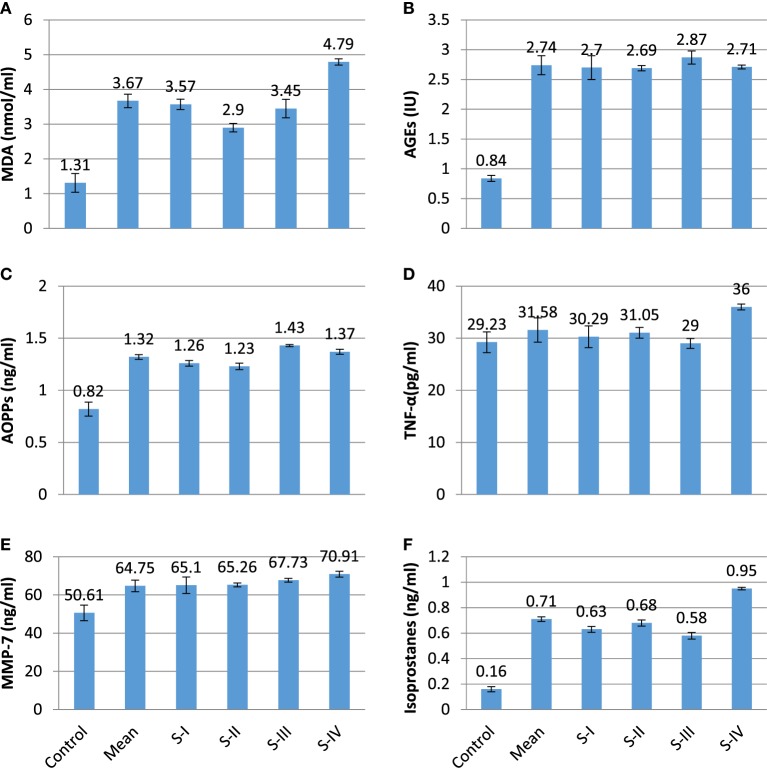
Profile of different prognostic variables in CA colorectal vs controls. **(A)** MDA (nmol/ml); **(B)** AGEs (IU); **(C)** advanced oxidation protein products (AOPPs) (ng/ml); **(D)** TNF-α (pg/ml); **(E)** matrix metalloproteinase-7 (MMP-7) (ng/ml); and **(F)** isoprostanes (ng/ml).

The present experimental work was designed to evaluate the role of oxidative changes in the development and progression of CRC. The results of all parameters are given in Table 1 and shown in Figures 1 and 2. ROS and reactive nitrogen species seem to play a very critical and destructive role in the development of the disease as reflected by the profile of enzymatic and non-enzymatic antioxidants specifically GSH (μg/dl), SOD (μg/dl), CAT (μmol/mol of protein), GRx (μmol/ml), and GPx (mmol/dl), respectively. All the aforesaid variables depicted very significant differences and also showed marked variation as the stage proceeds, represented in Figures 1 and 2, respectively. Significantly (*p* = 0.011, 0.001, 0.000, and 0.001) lower levels of GSH (4.86 ± 0.78 vs 9.65 ± 1.13), SOD (0.08 ± 0.012 vs 0.46 ± 0.017), CAT (2.45 ± 0.03 vs 4.22 ± 0.19), and GRx (5.16 ± 0.06 vs 7.23 ± 0.36) in diseased group were recorded as compared with control. All of them were highly significant. GSH showed increase in stage III and relative increase in stage IV as compared with stage II. The decreasing trend of SOD was revealed as the stage proceeds. CAT activity increases in the initial stages l and II then decreases in stage III, after that again increases in stage IV. As the disease progress, GRx first shows increasing trend than move toward the declining trend. In contrast to other stress markers, highly significant (*p* = 0.008) higher levels of GPx (6.64 ± 0.19 mmol/dl) were observed in the subjects in comparison with control group (1.58 ± 0.30 mmol/dl). The oxidative biomarker GPx elevated in stages l, II, and IV but decreased in stage III in CRC patients. Highly significant (*p* = 0.017, 0.034, and 0.016, respectively) decreased levels of vitamin A (0.81 ± 0.07 vs 2.37 ± 0.15 mg/ml), vitamin E (15.42 ± 1.26 vs 25.96 ± 2.19 mg/ml), and vitamin C (47.67 ± 7.69 vs 80.37 ± 10.21 mg/ml) were observed in patients in contrast to control group. The levels of vitamin A and vitamin E increased in stage I, II, and III but decreased in stage IV. Vitamin C first showed increasing trend than shows decline trend. The reversal of antioxidants in later stages of CRC may be due to compensatory mechanisms in cancerous cells. The levels of lipid peroxidation product, MDA (nmol/ml) was also assessed which shows significantly (*p* = 0.012) increased level in CRC patients as compared with control groups (3.67 ± 0.19 vs 1.31 ± 0.27). The increased levels at stage IV as compared with stage I, II, and III show more deterioration in stage IV in CRC patients. The levels of protein oxidation products [AGEs (2.74 ± 0.16 vs 0.84 ± 0.05 IU) and AOPPs (1.32 ± 0.02 vs 0.82 ± 0.07 ng/ml)] were significantly (*p* = 0.033 and 0.013, respectively) increased in subjects as compared with control. Both variables show increasing trend at all four stages in CRC patients, as damage proceeds. The levels of TNF-α were also assessed in diseased group in comparison with control group differed significantly (*p* = 0.019). All the four stages show minor difference as compared with control group. Furthermore, the levels of MMP-7 (64.75 ± 3.03 vs 50.61 ± 4.09 ng/ml) and isoprostanes (0.71 ± 0.03 vs 0.16 ± 0.02 ng/ml) were also analyzed, which bring about a positive relation in accordance with each other. Both the variables significantly showed highly significant (*p* = 0.037 and 0.027, respectively) increased levels in patient group in contrast to control group. MMP-7 and isoprostanes show increasing trend in all the four stages I, II, III, and IV, which shows metastasis.

**Table 1:** Profile of different variables having potential role in the development of colorectal cancer at different stages.

Variables	Control ± SD (*n* = 20)	Mean ± SD (*n* = 50)	Stage I (*n* = 24)	Stage II (*n* = 17)	Stage III (*n* = 7)	Stage IV (*n* = 2)	*p* < 0.05
MDA (nmol/ml)	1.31 ± 0.27	3.67 ± 0.19	3.57 ± 0.15	2.90 ± 0.12	3.45 ± 0.26	4.79 ± 0.09	0.012
Superoxide dismutase (μg/dl)	0.46 ± 0.017	0.08 ± 0.012	0.10 ± 0.013	0.12 ± 0.012	0.10 ± 0.07	0.09 ± 0.003	0.001
GSH (μg/dl)	9.65 ± 1.13	4.86 ± 0.78	4.95 ± 0.33	4.70 ± 0.45	5.03 ± 0.32	4.79 ± 0.45	0.011
Catalase (μmol/mol of protein)	4.22 ± 0.19	2.45 ± 0.03	2.41 ± 0.06	2.39 ± 0.07	2.31 ± 0.09	2.39 ± 0.08	0.000
TNF-α (pg/ml)	29.23 ± 1.99	31.58 ± 2.32	30.29 ± 2.09	31.05 ± 1.02	29.00 ± 0.95	36.00 ± 0.56	0.019
Matrix metalloproteinase-7 (ng/ml)	50.61 ± 4.09	64.75 ± 3.03	65.10 ± 4.32	65.26 ± 1.01	67.73 ± 0.99	70.91 ± 1.55	0.037
GRx (μmol/ml)	7.23 ± 0.36	5.16 ± 0.06	5.45 ± 0.08	5.37 ± 0.05	4.82 ± 0.03	4.21 ± 0.07	0.001
GPx (mmol/dl)	1.58 ± 0.30	6.64 ± 0.19	6.53 ± 0.09	6.58 ± 0.29	6.55 ± 0.11	6.90 ± 0.23	0.008
Advanced oxidation protein products (ng/ml)	0.82 ± 0.07	1.32 ± 0.02	1.26 ± 0.03	1.23 ± 0.03	1.43 ± 0.01	1.37 ± 0.02	0.013
AGEs (IU)	0.84 ± 0.05	2.74 ± 0.16	2.70 ± 0.20	2.69 ± 0.04	2.87 ± 0.11	2.71 ± 0.03	0.033
Isoprostanes (ng/ml)	0.16 ± 0.02	0.71 ± 0.03	0.63 ± 0.02	0.68 ± 0.04	0.58 ± 0.05	0.95 ± 0.01	0.027
Vit A (mg/ml)	2.37 ± 0.15	0.81 ± 0.07	0.77 ± 0.04	0.78 ± 0.09	0.93 ± 0.05	0.77 ± 0.06	0.017
Vit E (mg/ml)	25.96 ± 2.19	15.42 ± 1.26	14.81 ± 0.36	15.96 ± 0.66	16.14 ± 0.26	14.80 ± 0.17	0.034
Vit C (mg/ml)	80.37 ± 10.21	47.67 ± 7.69	51.14 ± 4.35	51.59 ± 3.55	48.71 ± 1.58	39.26 ± 2.01	0.016

## Discussion

This study showed that there is reduction in antioxidative status while increase in pro-oxidant levels in CRC (CRC) patients. As the stages of CRC proceed, the levels of different antioxidants slightly reverse which may be due to compensatory mechanisms in cancerous cells. On the other hand, marked increase in pro-oxidant levels was observed as the stage advances. Lower levels of SOD were discovered in CRC group, and it shows an inverse correlation with MDA (Table 2; SOD vs MDA, *r* = −0.643**). SOD first shows increasing trend in the initial stages than move toward the decline trend in later stages, the findings were in contradiction with the previous study (12). GSH is involved in the inhibition of free radicals induced carcinogenesis, but its levels were reduced in CRC patients and also showed slight reversal in stage III, this finding was inconsistent with a recent study showing increasing trend at the first two stages then decreases in the remaining stages (22).

**Table 2:** Pearson correlation coefficients of different variables of colorectal cancer in males.

Variables	GSH	Superoxide dismutase (SOD)	Catalase (CAT)	GRx	GPx	Vit A	Vit E	Vit C	MDA	AGEs	Advanced oxidation protein products (AOPPs)	TNF-α	Matrix metalloproteinase-7 (MMP-7)	Isoprostanes
GSH	1	0.235	0.195	0.594*	0.235	0.356	0.159	0.423	−0.559*	0.326	0.426	0.499	−0.399	−0.196
SOD		1	0.588*	0.341	0.166	0.331	0.499	0.341	−0.643**	−0.499	−0.411	−0.594*	−0.588*	−0.465
CAT			1	0.166	0.120	0.265	0.109	0.015	−0.544*	−0.523*	−0.165	−0.464	−0.326	−0.432
GRx				1	0.599*	−0.316	−0.321	−0.326	−0.501*	−0.356	−0.235	−0.475	−0.326	−0.162
GPx					1	0.432	0.319	0.112	0.421	0.395	0.465	0.316	0.552*	0.425
Vit A						1	0.192	0.331	−0.254	−0.235	−0.235	−0.165	−0.229	−0.329
Vit E							1	0.195	−0.562*	−0.058	−0.165	−0.326	−0.561*	−0.366
Vit C								1	−0.795***	−0.199	−0.119	−0.195	−0.195	−0.444
MDA									1	0.523*	0.499	0.585*	0.465	0.846***
AGEs										1	0.651**	0.492	0.648**	0.452
AOPPs											1	0.666**	0.495	0.399
TNF-α												1	0.624**	0.769***
MMP-7													1	0.765***
Isoprostanes														1

*p < 0.05, **p < 0.01, ***p < 0.001.

Beta carotene, a pro-vitamin A carotenoid deficiency not only favors polyp dysplasia leading to CRC development but also associated with poor absorption of vitamin E, resulting in its deficiency leading to oxidative insult causing upregulated intracellular signaling which mediates tumor growth (23, 24). Vitamin C plays a key role in the production of collagen and its deficit upsets the integrity of intracellular matrix and promotes tumor proliferation. Heine-Broring et al. (25) suggested an inverse correlation between risk of CRC and deficiency of vitamins A, E, and C, similar findings were observed in this study. On the other hand, recently, no association was found between supplementation of these dietary vitamins and risk of CRC (26). Malondialdehyde, a highly toxic aldehyde is the end product of lipid peroxidation. In CRC, the reduction in SOD and vitamin C levels results in lipid peroxidation which eventually increases the levels of MDA (SOD vs MDA, *r* = −0.643** and vitamin C vs MDA, *r* = −0.795***), in accordance with previous studies (12, 27). It has been revealed that AOPPs, the dityrosine containing protein cross-linking products formed by the attack of ROS on albumin and are increased significantly in colorectal carcinoma (10), similar findings were seen in this study.

**Figure 3:**
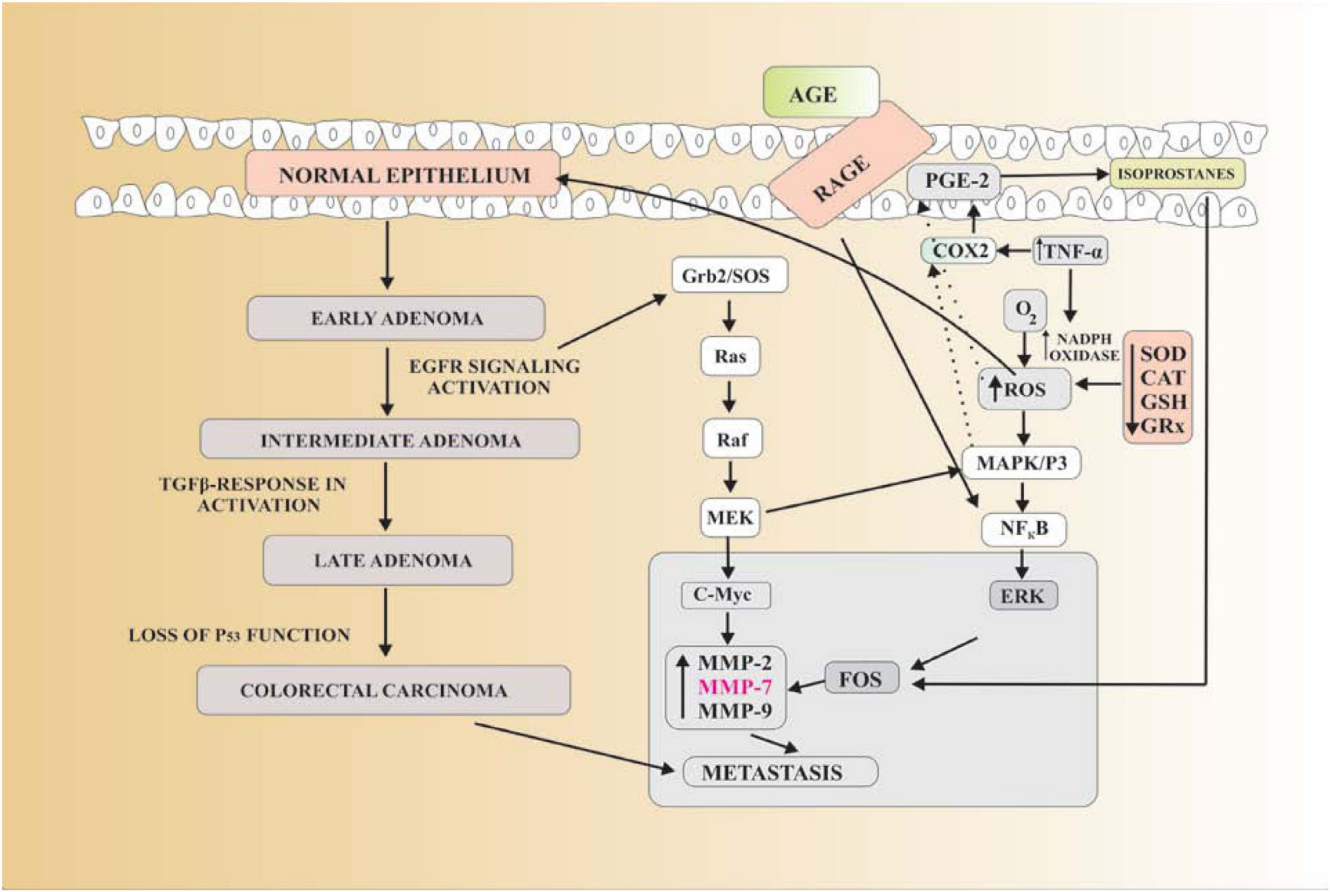
Reactive oxygen species (ROS) produce oxidative stress which in return converts the normal epithelium to early adenoma which leads to the next stage that is intermediate adenoma by the activation of EGFR signaling. Tumor growth factor-β inactivation further leads to the late adenoma. Due to loss of p53 function late adenoma is converted into colorectal carcinoma. P53 is a gene that inhibits the mutagenesis. Due to their lost function, the chance of colorectal cancer (CRC) is increased. EGFR signaling activation stimulates the Grb2/SOS protein which further activates the Ras, Raf, and MEK pathways. It further stimulates the two pathways. In first pathway, MEK activates the c-Myc gene which upregulates matrix metalloproteinase-7 (MMP-7), causing metastasis. In second pathway, Ras–Raf–MEK pathway activates MAPK/P3 which in turn upregulates FOS protein, also causes MMP-7-dependent metastasis. AGE-RAGE complex activates NF-κB which in turn also activates FOS protein-mediated MMP-7 upregulation. Decreased antioxidants and increased TNF-α result in enhanced ROS production which not only activates MAPK/P3 but also increases in prostaglandin E-2 (PGE-2) by stimulating cyclo-oxygenase-2 (COX2). ROS attack PGE-2 in cell membrane to produce isoprostanes, which again increase FOS resulting in MMP-7 production leading to metastasis of CRC.

Matrix metalloproteinase-7 expression is linked to the invasion and metastasis of cancer cells by disrupting the basement membrane, as epithelial cells of large intestine mainly show MMP-7 expression which depicts formation of adenoma from normal colorectal mucosa thus, considered as a prognostic factor (9). Mostly, MMPs secreted as inactive zymogens and are stimulated by pro-oxidants and inflammatory markers while inhibited by tissue inhibitors matrix metalloproteinases (TIMPS) (28). Higher levels of TNF-α cause enhanced activation of cyclo-oxygenase-2 results in increased production of prostaglandin E-2 (PGE-2) which under the effect of ROS mediate isoprostanes synthesis leading to upregulation of MMPs as shown in Figure 3, and same findings were previously reported by Chu et al. (29). This study shows positive correlation between TNF-α and isoprostanes (TNF-α vs isoprostanes, r = 0.769***). Tumor necrosis factor develops tumor invasion in CRC through c-Src oncogene activation, as it promotes angiogenesis, proliferation, and metastasis (30). Isoprostanes are prostaglandins like complexes, which are formed by the action of free radicals on arachidonic acid in the presence of an enzyme cyclo-oxygenase. This study also shows a very strong positive correlation of isoprostanes with MMP-7 (Isoprostanes vs MMP-7, *r* = 0.765***), previously same findings were observed by Myfanwy et al. (31).

## Conclusion

This study concludes that there is reduction in antioxidative status and increase in pro-oxidants levels in CRC patients. Oxidative stress-mediated MMPs and isoprostanes are innovative and prognostic biomarkers of CRC proliferation. Early detection of MMPs and other stress biomarkers can hamper the development and progression of CRC. Thus, therapeutic strategies (tissue inhibitor matrix metalloproteinase I and II) can be used by the clinicians to limit the proliferation and metastasis of CRC.

## Ethics Statement

This study was carried out in accordance with the recommendations of Center for research in Molecular Medicine (CRiMM), The University of Lahore, Research Ethical Committee of the Institute of Molecular Biology and Biotechnology (IMBB). The protocol was approved by the Research Ethical Committee of the Institute of Molecular Biology and Biotechnology (IMBB). All subjects gave written informed consent in accordance with the Declaration of Helsinki.

## Author Contributions

MR, AM, MJ, and MA-Q: conceived and designed the manuscript. MABA, SZ, AQ and NH: conducted experiment. MA, SW, SS, and MN: written the manuscript. OAB, MZ, HC: reviewed the manuscript, AAG, MAZ, AA-G and MSJ: evaluated the manuscript.

## Conflict of Interest Statement

The authors declare that the research was conducted in the absence of any commercial or financial relationships that could be construed as a potential conflict of interest.
